# Vaccination with recombinant *Brugia malayi* cystatin proteins alters worm migration, homing and final niche selection following a subcutaneous challenge of Mongolian gerbils (*Meriones unguiculatus*) with *B. malayi* infective larvae

**DOI:** 10.1186/1756-3305-7-43

**Published:** 2014-01-22

**Authors:** Sridhar Arumugam, Bin Zhan, David Abraham, Danielle Ward, Sara Lustigman, Thomas R Klei

**Affiliations:** 1 Department of Pathobiological Sciences, LSU School of Veterinary Medicine, Louisiana State University, 1909 Skip Bertman Drive, Baton Rouge, LA 70803, USA; 2 Department of Pediatrics and Tropical Medicine, Baylor College of Medicine, Texas Children Hospital, 1102 Bates St, Ste.550, Houston, TX 77030, USA; 3 Department of Microbiology and Immunology, Kimmel Cancer Center, Thomas Jefferson University, 530 BLSB, 233 S. 10th Street, Philadelphia, PA 19107, USA; 4 Laboratory of Molecular Parasitology, Lindsley F. Kimball Research Institute, New York Blood Center, 310 E 67th St, New York, NY 10065, USA

**Keywords:** Brugia malayi, Cysteine protease inhibitors, Filariasis, Vaccination, Worm migration, Immunomodulation

## Abstract

**Background:**

Cysteine protease inhibitors of *Brugia malayi* have been ascribed to be involved in parasite development as well as to immunomodulate the host’s immune response. In *Onchocerca volvulus*, Onchocystatin has been shown to induce partial protection in the mouse diffusion chamber vaccination model. In the present study we investigated the impact of vaccination with recombinant *Bm*-CPI-1 and *Bm*-CPI-2 proteins on protection against a subcutaneous challenge of *B. malayi* third stage larvae in gerbils.

**Findings:**

Vaccination with *E. coli* derived recombinant *B. malayi* cysteine protease inhibitors (*Bm*-CPI-1 or -2) did not confer protection against *B. malayi* L3 challenge infection in gerbils but altered the homing of a significant number of adult worms from the lymphatics to the heart and lungs.

**Conclusion:**

*Bm*-CPI vaccination-induced alteration in worm migration is consistent with our previous observations in gerbils vaccinated with *B. pahangi* excretory-secretory (ES) proteins, which resulted in delayed migration of the L3s and altered the final location of adult worms. Similar observations have also been made in dogs vaccinated with *Ancylostoma caninum* proteins; an increased number of worms were recovered in the colon and not the expected small intestine. A change in the final niche was also reported in immune versus non-immune hosts of two other gut dwelling nematodes. Vaccination induced alteration of the parasite’s final homing might be a rare or a common phenomenon, which unfortunately is rarely recorded. The reason for the alteration in the final niche selection by adult nematode worms following vaccination is unknown and necessitates further investigation.

## Findings

### Background

An array of filarial vaccine candidates have been tested in various rodent models including the *Brugia malayi* Mongolian gerbil system. These have been reviewed recently by Morris *et al*. [1]. Vaccination with *B. malayi* irradiated L3 larvae conferred the highest level of protection (56 to 91%) against subcutaneous (SC) or intraperitoneal (IP) L3 challenge [1]. Apart from irradiated L3 larvae, soluble extracts of microfilariae (MF) and adult worms and some recombinant protein antigens conferred significant protection against L3 challenged rodent animal models. The *B. malayi* abundant larval transcript I (Bm-ALTI) showed the highest levels of protection of 76% [1]. However, so far no vaccine based strategy has demonstrated complete protection against a challenge infection in any permissive animal model. This is likely due to the complexity of filarial infections and the ability of filarial parasites to modulate the immune system to increase their longevity in the mammalian host [2,3]. *B. malayi*, filarial infection in humans is characterized by an antigen-specific unresponsiveness in the peripheral T cell populations of chronically infected patients [4,5]. Filarial parasite secreted proteins can dampen the host immune response and immunopathology may occur from a dysregulated response to infection [3]. Excretory-secretory (ES) proteins of filarial nematodes play a major role in pathogenesis, immunodiagnosis of helminth infections and host immune regulation [6,7]. The major set of proteins secreted by filarial nematodes includes proteases, protease inhibitors, venom allergen homologues, glycolytic enzymes and lectins [8,9].

Cystatins or cysteine protease inhibitors (CPIs) have been described across the animal and plant kingdoms. The role of CPIs in parasitic nematodes has been attributed to essential developmental processes and to specific interactions with the parasite’s vector and/or mammalian hosts [10,11]. In *B. malayi*, three cysteine protease inhibitors have been characterized; these are *Bm*-CPI-1, *Bm*-CPI-2 and *Bm*-CPI-3 [10,12,13]. *Bm*-CPI-2 has been found in ES products and is expressed in all life stages of *B. malayi. Bm*-CPI-2 acts as an immunomodulator by blocking the activity of mammalian proteases including the antigen-processing enzyme asparaginyl endopeptidase, whereas Bm-CPI-1 and -3 are expressed in L2 and L3 stages and have been described to have functions in the mosquito vector necessary for transmission of the parasite [10,13]. The amino acid sequences identified between Bm-CPI-1 and Bm-CPI-2 is 28% and the sequence alignment is shown in Additional file 1: Figure S1.

In *O. volvulus*, a cysteine protease inhibitor, onchocystatin (Ov7; Ov-CPI-2) was described by Lustigman *et al*. [14]. The recombinant onchocystatin administered with alum as an adjuvant was able to induce 49% reduction in worms in an L3 challenge within diffusion chambers [15,16]. Moreover, the levels of anti-Ov-CPI-IgG3 cytophilic antibodies were elevated in the putatively immune (PI) and significantly increased with age in infected individuals implicating a potential role of *Ov*-CPI-2 in the protective immunity in humans [17]. In the *Litomosoides sigmodontis*-mouse model, *L. sigmodontis* cysteine protease inhibitor-2 (*Ls*-CPI-2) acts as an immunomodulator and DNA vaccination with mutated *Ls*-CPI-2 along with mutated *L. sigmodontis* abundant larval transcript-1 (*Ls*-ALT-1) conferred significant protection against an L3 challenge leading to reduced adult worm burden and a reduction in peripheral microfilaremia [18,19]. In the current study, we measured the effect of vaccination with recombinant *Bm*-CPI-1 or *Bm*-CPI-2 on protective immunity to *B. malayi* infection in gerbils following a SC challenge of L3. Our results showed that vaccination with *Bm*-CPI-1 and *Bm*-CPI-2 when formulated in alum did not confer protection against *B. malayi* infection in Mongolian gerbils. However, it affected the final niche where the adult worms resided. Significantly more worms were found in the heart and lungs and fewer worms were found in the lymphatics of both *Bm*-CPI-1 and *Bm*-CPI-2 vaccinated animals in comparison to the adjuvant controls. To the best of our knowledge, this is the first report to demonstrate that vaccination with recombinant filarial proteins affects filarial parasite adult worm migration and selection of their final niche.

## Methods

### Expression of recombinant *Bm*-CPI-1 and *Bm*-CPI-2

DNAs encoding for the *Bm*-CPI-1 (GenBank accession # AF177192) and *Bm*-CPI-2 (GenBank accession #AF015263) plus a 6-histidine-tag at C-terminus were synthesized by GenScript (Piscataway, NJ) and subsequently subcloned in-frame into the *E. coli* expression vector pET41a (Novagen). The recombinant plasmids were transformed into BL21 (DE3) (Novagen) and recombinant proteins were induced with 0.5 mM IPTG and purified with Ni-column as described [20]. Putative endotoxin, LPS, was removed from the recombinant proteins using ToxinEraser endotoxin removal kit following the manufacturer’s protocol (GenScript, Piscataway, NJ).

### Animals, vaccination and challenge with L3s

Eight week old male Mongolian gerbils (*Meriones unguiculatus*) purchased from Charles River Laboratories (Wilmington, MA) were maintained on standard rodent chow and water *ad libitum*. The Institutional Animal Care and Use Committee (IACUC) at Louisiana State University (LSU) approved the animal experimental protocols. *B. malayi* L3s were recovered from infected *Aedes aegypti* mosquitoes using the previously described Baermann technique [21]. For vaccine challenge experiments, 100 *B. malayi* L3s in 0.5 ml of RPMI were injected subcutaneously into the inguinal region of the left leg.

For proper formulation with alum, the complete binding of *Bm*-CPI-1 and *Bm*-CPI-2 to alum (Rehydragel LV, General Chemical, NJ) was tested. Briefly, 2.5 μg of either recombinant *Bm*-CPI-1 or *Bm*-CPI-2 was mixed with 32 μg of alum for 30 min at room temperature with shaking, centrifuged at 2000 rpm for 5 min and then the supernatant was run on a 12% SDS-PAGE gel. There were no bands observed (Additional file 2: Figure S2), suggesting that both recombinant proteins efficiently bind to alum using this protein:adjuvant ratio.

Recombinant *Bm*-CPI-1 or *Bm*-CPI-2 was formulated with alum using the same ratio for vaccination of gerbils undergoing the intramuscular (IM) route of immunization. The following groups of male gerbils (N = 10; 8 week old) were vaccinated with: 1) 25 μg of *Bm*-CPI-1 absorbed to 320 μg of alum in TBS; 2) 25 μg of *Bm*-CPI-2 absorbed to 320 μg of alum in TBS; 3) 320 μg of alum in TBS. The first vaccination (V1) was followed by two boosters at two week intervals (V2, V3). Pre-immune serum was collected 1 week before the start of each experiment and designated as PI and 1 week after V3; designated Post-V3. Two weeks after the third vaccination, each gerbil was challenged with 100 L3s of *B. malayi* subcutaneously (SC) in the medial surface of the left leg.

Necropsy of all gerbils was performed 43 days post-challenge. Right and left popliteal lymph nodes, right and left renal lymph nodes, ilio-lumbar vessels, right and left spermatic cord lymphatics, right and left sub-inguinal and iliac lymph nodes, and right and left testes were gently teased in PBS under a stereomicroscope. In addition, the peritoneal cavity was washed with 1× PBS. The viscera and the carcass were soaked for 1 hour in 1× PBS. Later the heart and lungs were gently teased in 1× PBS. Following teasing, all tissues were left to soak for 1 hour to allow worms to emerge. The worms were recovered, counted, and then stored in 70% ethanol and 30% glycerin for observation if needed. For the purposes of discussion, we refer to worms collected from all lymphatic organs, testis and spermatic cords, as worms in the lymphatics, and worms collected from the heart and lungs as worms in heart & lungs.

### Measuring IgG response against *Bm*-CPI-1 and *Bm*-CPI-2 by ELISA

To detect gerbil-specific IgG responses we raised polyclonal anti-gerbil IgG antibodies in rabbits. Briefly, total gerbil serum proteins were extracted by ammonium sulfate precipitation followed by the purification of the total IgG using the Pierce protein G IgG plus orientation kit and the manufacturer’s instructions (Thermo Scientific, IL). Polyclonal anti-sera against gerbil IgG in rabbits was raised under a contract with Pacific Immunology, CA. The gerbil IgG specific antibody response induced by vaccination with recombinant *Bm*-CPI-1 or *Bm*-CPI-2 was measured using the Rabbit Serum Antibody detection ELISA kit (Alpha Diagnostic, San Antonio, TX). Briefly, each well of a 96 well microplate was coated at 4°C overnight with 100 μl of 1 μg/ml of the antigen (recombinant *Bm*-CPI-1 or *Bm*-CPI-2) in coating buffer. The plates were washed 3 times with 200 μl of 1× wash buffer, blocked with 200 μl of blocking solution at room temperature for 4 hrs, then after an additional 3 washes incubated for 2 hrs at room temperature with serial dilutions of the gerbil sera collected during the course of vaccination with either *Bm*-CPI-1, *Bm*-CPI-2 or alum alone. Following incubation of sera, the plates were washed 3 times with wash buffer and incubated with 100 μl of 1:5000 dilution of the rabbit anti-gerbil IgG for 1 hr. The plates were then washed 3 times with wash buffer and incubated with 100 μl of 1:5000 dilution of HRP-conjugated anti-rabbit IgG for 30 minutes followed by an additional 4 washes and the addition of 100 μl of TMB substrate for 15 minutes at room temperature. The reaction was stopped by adding 100 μl of stop solution (H_2_SO_4_) and the microplate was read at 450 nm using an ELISA reader (Molecular Devices, Sunnyvale, CA).

To check if sera from the *Bm*-CPI-1 vaccinated gerbils cross-reacted with the *Bm*-CPI-2 protein, we analysed *Bm*-CPI-2 coated plates with sera from *Bm*-CPI-1 vaccinated gerbils. Likewise *Bm*-CPI-1 coated plates were tested with sera from *Bm*-CPI-2 vaccinated gerbils.

### Statistics

Differences in worm recovery were analysed by the unpaired t test using GraphPad Prism version 4.03 for Windows (GraphPad Software, San Diego, California). Significance was when P ≤ 0.05.

## Results

Vaccination with recombinant *Bm*-CPI-1 and *Bm*-CPI-2 did not confer protection against *B. malayi* infection but altered the final niche selection by the adult worms.

**Figure 1:**
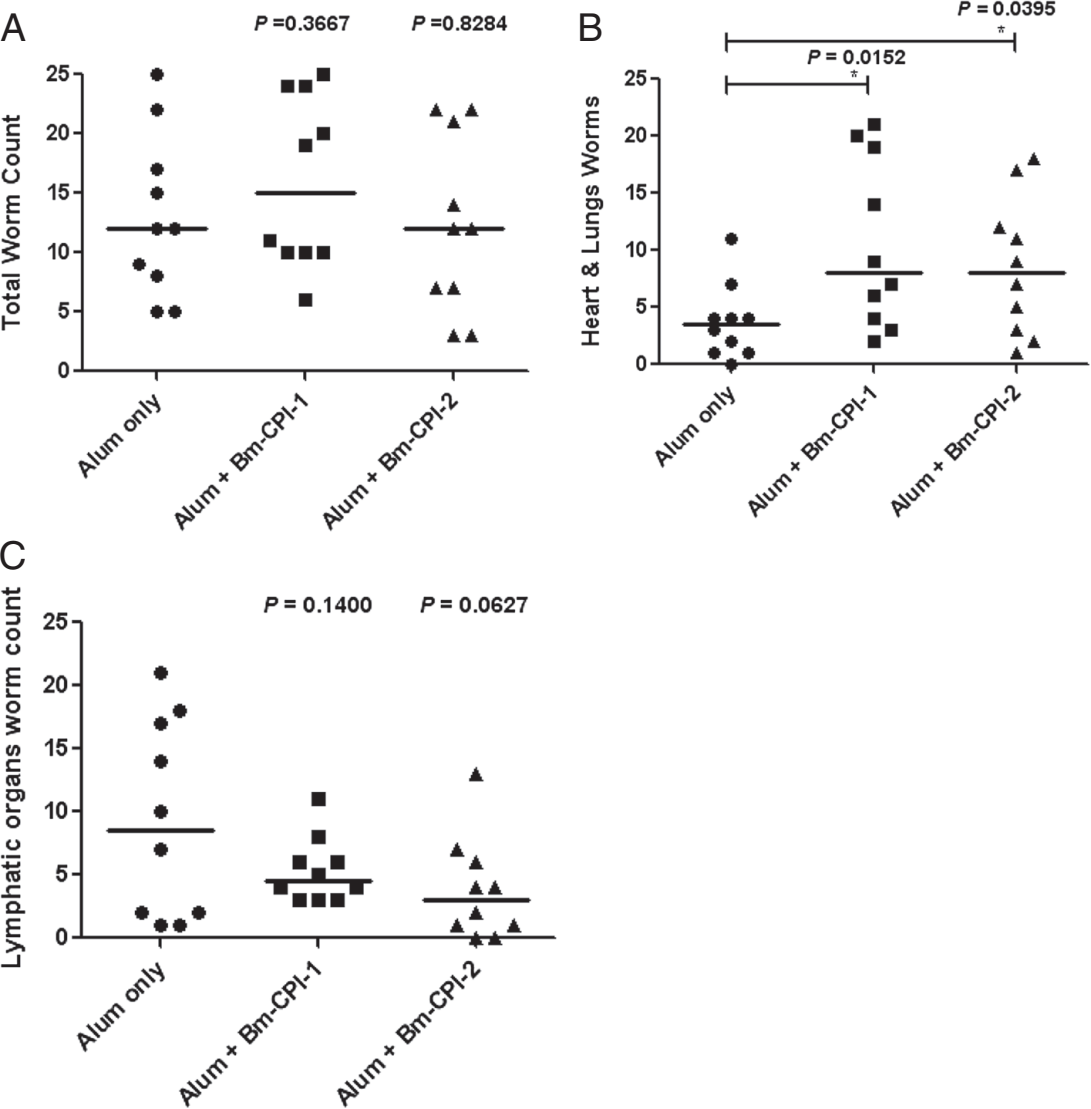
**Vaccination with recombinant *Bm***-**CPI**-**1 and *Bm***-**CPI**-**2 does not confer protection against *B*. *malayi *infection in Mongolian gerbils but affects migration of worms and habitat selection. A)** Total worm count. **B)** Distribution of worms in heart and lungs. **C)** Distribution of worms in lymphatic organs. Statistical significance was determined by unpaired t test using GraphPad Prism version 4.03, * denotes a significant difference between vaccinated group and alum adjuvant control, *P* ≤ 0.05. The line represents the median value.

Mongolian gerbils were vaccinated IM three times with recombinant *Bm*-CPI-1 or *Bm*-CPI-2 in alum before they were challenged with 100 *B. malayi* L3s SC. Forty-three days post-infection, the gerbils were euthanized and necropsy was performed; adult *B. malayi* worms were recovered from different tissues of gerbils and counted. Neither vaccination with recombinant *Bm*-CPI-1 nor *Bm*-CPI-2 resulted in significant protection against *B. malayi* infection compared to the adjuvant control group (Figure 1A). However, there was a difference in the tissue distribution of adult worms between the antigen vaccinated groups and the adjuvant control group. More worms were found in the heart and lungs and fewer worms were found in the lymphatic organs of gerbils vaccinated with recombinant *Bm*-CPI-1 and *Bm*-CPI-2 (Figure 1B and C). The percentages of worms in the heart and lungs of *Bm*-CPI-1 and Bm-CPI-2 vaccinated groups were 66% and 69% respectively, and the percentage of worms in the lymphatics of *Bm*-CPI-1 and *Bm*-CPI-2 vaccinated groups was 34% and 31%, respectively. The percentage of worms in the heart and lungs or the lymphatics in the alum control gerbils was 29% and 71%, respectively.

### Gerbil IgG response to *Bm*-CPI-1 and *Bm*-CPI-2 vaccination

The end point dilution of antigen specific IgG responses induced after three vaccinations were measured by ELISA. High end point dilution of antigen specific IgG responses (up to 1:2,048,000) were detected in serum from gerbils vaccinated with *Bm*-CPI-1, whereas no *Bm*-CPI-1 specific IgG was observed in pre-immune and alum control serum. Antigen specific IgG end point dilution of up to 1:512,000 was detected in serum from gerbils vaccinated with *Bm*-CPI-2. When the cross-reactivity between the antibody responses was analysed, an end point dilution of 1:512, 000 was detected when *Bm*-CPI-1 was tested with serum from gerbils vaccinated with *Bm*-CPI-2. Similarly, an end point dilution of 1:2,048, 000 was found when *Bm*-CPI-2 coated plates were tested with serum from gerbils vaccinated with *Bm*-CPI-1. The *Bm*-CPI-1 antibody end point dilution was slightly higher than the *Bm*-CPI-2.

## Discussion

Many investigators have reported protection in rodent-*Brugia* models against L3 infection with a large number of different recombinant *B. malayi* proteins [1]. However, there are no previous vaccination experiments reported that tested the efficacy of *Bm*-CPI-1 or *Bm*-CPI-2 in this model. Many studies also implicated the potential role of antibody-mediated killing of L3 as an effector mechanism [1]. While in our present study vaccination induced a significant antigen-specific IgG antibody response against *Bm*-CPI-1 and *Bm*-CPI-2, no protection against SC L3 infection was observed. It is important to note that unlike most other reported *B. malayi*-gerbil vaccination experiments, the challenge of L3s was administered SC, which is a more similar and natural route of infection in the human host. It also necessitates significant tissue migration by L3s and L4s in the host and final niche selection by the adult worms [22]. Migration and homing does not happen following an IP challenge or by using L3s implanted in diffusion chambers. These have been the most common routes of challenges used in the majority of the *B. malayi*-gerbil vaccination experiments reported. The current observations suggest that using our particular immunization regimens with both *Bm*-CPIs were not sufficient to block the establishment and development of *B. malayi* L3s in this model. It is possible that the appropriate protective immune responses were not generated by this vaccination.

Notably, although vaccination with *Bm*-CPI-1 and *Bm*-CPI-2 was not protective against *B. malayi* infection, it resulted in alteration of *B. malayi* larval migration and the final selection of the habitat where the adult worms resided. In the current experiment, the distribution of worms in the heart and lungs and in the lymphatics was shifted in such a way that more worms were recovered in the heart and lungs than in the lymphatics in comparison to alum control gerbils. The percentages of worms in the heart and lungs of *Bm*-CPI-1 and Bm-CPI-2 vaccinated groups was 66% and 69% respectively, and the percentage of worms in the lymphatics of *Bm*-CPI-1 and *Bm*-CPI-2 vaccinated groups was 34% and 31% respectively. The percentage of worms in the heart and lungs and in the lymphatics of alum control gerbils was 29% and 71% respectively. This is the first time out of 20 other experiments performed in our laboratory using 10 other recombinant proteins that such an outcome was observed. Over 20 experiments, the average percentage of worms recovered from the heart and lungs of alum control groups and the antigen-vaccinated groups was 42 and 44%, respectively. In comparison, the average percentage of worms recovered from the lymphatics and lymph nodes of alum control groups and antigen-vaccinated groups was 58 and 56%, respectively (Unpublished data). The recombinant *B. malayi* proteins used in these experiments are homologs of *O. volvulus* proteins reviewed elsewhere [23]. These proteins are *E. coli* expressed *Bm*-ALT-1 (*B. malayi* abundant larval transcript-1), *Pichia* expressed *Bm*-ALT-1, *E. coli* expressed *Bm*-ALT-2, *E. coli* expressed *Bm*-FAR-1 (*B. malayi* fatty acid retinol binding protein), *Pichia* expressed *Bm*-FAR-1, *E. coli* expressed *Bm*-FAR-2, *E. coli* expressed *Bm*-103 (*B. malayi* cDNA clone 103), *Pichia* expressed *Bm*-103, *E. coli* expressed *Bm*-RAL-2 (*B. malayi* novel protein reactive against larvae antiserum-2) and *Pichia* expressed *Bm*-RAL-2. The efficacy of these proteins in inducing a protective immunity will be discussed in subsequent publications.

The reasons for this altered migration and final location of the developed adult worms are not clear. Nor is the reason that this is only seen following vaccination with Bm-CPIs and not other recombinant proteins. Interestingly, in previous studies using the *B. pahangi*-gerbil model, we found that L3s induce an early acute inflammatory response that is modulated once the parasites are established in the lymphatics [24]. *Bm*-CPI-2 is a secreted protein and was shown to be immunomodulatory [13]. It is possible that the native *Bm*-CPI-2 secreted by L3s could reduce this initial acute inflammatory response against L3s and thus support the final residence of the worms in the lymphatics of control animals. However, as the vaccination with *Bm*-CPI-1 and/or CPI-2 induced a strong antibody-mediated response, it is possible that these strong responses have blocked the function of the native *Bm*-CPI-2 in the immunized gerbils. Moreover, as antibodies to *Bm*-CPI-1 and -2 proteins cross-react with each other it is possible that vaccinations with Bm-CPI-1 have resulted in similar outcomes. As a result, a non-modulated inflammatory response in this instance may not have been able to kill L3s, but rather created an unfavourable environment for the larvae in the lymphatics which altered their behaviour shunting them and subsequent stages away from the lymphatics to the heart and lungs. Our current findings are closely related to those of a recently reported study in the *B. pahangi*-gerbil model where vaccination of gerbils with *B. pahangi* ES secreted during the first 24 hours of culture slowed the early migration of the L3 challenge and subsequently also affected the worm’s migration from the lymphatics on the right side of the host to those on the left side [25]. Altered homing of adult parasites following vaccination has also been reported in dogs after vaccination with recombinant *Ancylostoma caninum* tissue inhibitor of metalloprotease (Ac-TMP) compared to dogs vaccinated with alum alone. The vaccination with recombinant proteins resulted in the reduction of the number of adult hookworms recovered from the small intestine and a concomitant increase in the number of adult hookworms recovered from the colon [26]. The effects of host immunity on parasite behavior have been reviewed by Damian RT [27]. The migration of *Schistosoma mansoni* and *Nippostrongylus brasiliensis* in mice like that of *B. pahangi* in gerbils was much reduced in the immune hosts. Similar to the report on *A. caninum*, the migration of larvae and adult worms of *N. dubius* and the adult worms of *Trichostrongylus colubriformis* in guinea pigs were significantly altered in the immune hosts.

Notably, the literature of vaccination experiments using filarial parasite-rodent models have so far and understandably focused on analyzing the reduction in worm burden in vaccinated groups. Many if not most of these experiments utilized IP or diffusion chamber L3 challenges. Following an IP challenge, most worms do not leave the peritoneal cavity and have no need to migrate to find an appropriate habitat for development. Most of the other experiments using the SC route of challenge did not report on adult worm location at necropsy. We present data here which demonstrates the effect of vaccination with recombinant protein and presumably the resulting immune response on the filarial worm’s migration and their final selection of habitat. Alterations of a parasite’s final niche after vaccination may be a rare phenomenon or a common phenomenon that is rarely reported. However, this can only be demonstrated in animal models using permissive hosts and challenge systems that allow for more natural migratory patterns. The reason for alteration in the migratory pattern of *Brugia* adult worms following vaccination is unclear. While speculative, Damian [27] suggested several possible explanations as to why this may occur which include: immune or inflammatory factors present in immune animals blocking normal chemoreceptors, the disruption of chemotactic gradients, or the modification of the local environment. All of these may explain in part our observations, however, why we observed this only following vaccination with *Bm*-CPIs and not with other filarial vaccine candidates is perplexing. Clearly the understanding of this phenomenon necessitates further investigation.

## Conclusion

Vaccination with recombinant *Bm*-CPI-1 and *Bm*-CPI-2 in alum induced a significant antibody response to both proteins and antibodies to these proteins cross-reacted with each other. However, vaccination did not protect gerbils against *B. malayi* infection in terms of worm reduction. Vaccination did alter the worm distribution resulting in a decrease of adult worms in lymphatic tissues and a significant increase in worm migration to the heart and lungs. The reasons for the alteration in migration and establishment of worms in *Bm*-CPI-1 and *Bm*-CPI-2 vaccinated gerbils are unknown. Beside the main focus on reduction in worm burden in vaccination experiments in the filarial parasites-animal model described so far in literature, we have recognized a unique and rarely reported phenomenon on the effect of vaccination on filarial worm migration and habitat selection.

## Abbreviations

Bm-CPIs: *Brugia malayi* cysteine protease inhibitors; ES: Excretory-secretory products; SC: Subcutaneous; IP: Intraperitoneal; IM: Intramuscular; MF: Microfilariae.

## Competing interests

The authors declare that they have no competing interests.

## Authors’ contributions

TRK, SL, SA and DA conceived the idea and designed the experiments. SA performed the experiments, analyzed the data and wrote the paper. BZ performed expression and purification of recombinant proteins and cysteine protease inhibition assay. DW raised *B. malayi* L3 larvae and assisted in gerbil necropsy. All authors read and approved the final version of the manuscript.

## Supplementary Material

Additional file 1: Figure S1 Amino acid sequence alignment between Bm-CPI-1(GenBank accession number: XP_001895476) and Bm-CPI-2 (AF177193_1). Sequences were aligned using CLUSTAL W and prepared for display by BOXSHADE. The identical amino acids are shaded in black and similar substitution in gray. Amino acids common to every sequence are marked by asterisk below the alignment. The percentage of sequence identity between Bm-CPI-1 and Bm-CPI-2 is shown at the end of Bm-CPI-1 sequence. Click here for file

Additional file 2: Figure S2 Absorption of recombinant Bm-CPI-1 and Bm-CPI-2 on alum. 2.5 μg of recombinant Bm-CPI-1 or BmBm-CPI-2 was incubated with 32 μg of alum for 30 min. After centrifugation at 2000 rpm for 5 min, the supernatant was loaded on a 14-20% SDS-PAGE gel. M, molecular weight marker; Lane 1, 2.5 μg Bm-CPI-1; Lane 2, supernatant from alum absorbed Bm-CPI-1; Lane 3, 2.5 μg Bm-CPI-2.; Lane 4, supernatant from alum absorbed Bm-CPI-2. Click here for file
