# Supplementary figures and images for: Vaccination with recombinant Brugia malayi cystatin proteins alters worm migration, homing and final niche selection following a subcutaneous challenge of Mongolian gerbils (Meriones unguiculatus) with B. malayi infective larvae
Source: Parasit Vectors. 2014 Jan 22;7:43. doi: 10.1186/1756-3305-7-43 (PMC3910689; doi:10.1186/1756-3305-7-43)

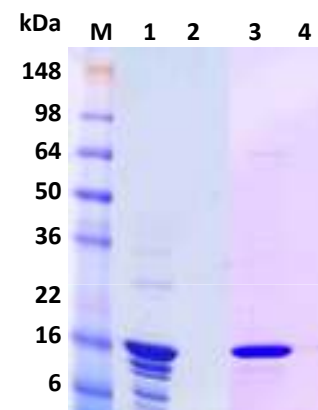

Suppl. Fig. S2

Supplement: Additional file 2: Figure S2 — Absorption of recombinant Bm-CPI-1 and Bm-CPI-2 on alum. 2.5 μg of recombinant Bm-CPI-1 or BmBm-CPI-2 was incubated with 32 μg of alum for 30 min. After centrifugation at 2000 rpm for 5 min, the supernatant was loaded on a 14-20% SDS-PAGE gel. M, molecular weight marker; Lane 1, 2.5 μg Bm-CPI-1; Lane 2, supernatant from alum absorbed Bm-CPI-1; Lane 3, 2.5 μg Bm-CPI-2.; Lane 4, supernatant from alum absorbed Bm-CPI-2. [file 1756-3305-7-43-S2.pdf]
