# Supplementary material for: Vaccination with recombinant Brugia malayi cystatin proteins alters worm migration, homing and final niche selection following a subcutaneous challenge of Mongolian gerbils (Meriones unguiculatus) with B. malayi infective larvae
Source: Parasit Vectors. 2014 Jan 22;7:43. doi: 10.1186/1756-3305-7-43 (PMC3910689; doi:10.1186/1756-3305-7-43)
Supplement: Additional file 1: Figure S1 — Amino acid sequence alignment between Bm-CPI-1(GenBank accession number: XP_001895476) and Bm-CPI-2 (AF177193_1). Sequences were aligned using CLUSTAL W and prepared for display by BOXSHADE. The identical amino acids are shaded in black and similar substitution in gray. Amino acids common to every sequence are marked by asterisk below the alignment. The percentage of sequence identity between Bm-CPI-1 and Bm-CPI-2 is shown at the end of Bm-CPI-1 sequence. [file 1756-3305-7-43-S1.pdf]

```
Bm-CPI-1    1 ----REIRLSR---------------ATYNEDDEEIQEVAEKAMEQVNDQTRYRNLYKLV
Bm-CPI-2    1 LIHRREIPHMESKGQMQRGQVLLGGWQERSPEDNEILELLPSVLTKVNQQSNDEYHLMPI
                  ***                        * ** *          ** *

Bm-CPI-1   42 RVISAQTQVVAGIKYYLTILAAPTTCRKGAVG-MNPMKCTIDSSKPTKQFKIEVWSAPWQ
Bm-CPI-2   61 KLLKVSSQVVAGVKYKMEVQVARSECKKSASEQVNLKTCKKLEGHPDQVMTLEVWEKPWE
                     ***** **       *   *   *    *   *      *      ***  **
                                    identity
Bm-CPI-1  101 NTFKVTLT--------         28%
Bm-CPI-2  121 DFLQVNILETKVLSSV
                  *
```

Suppl Fig. S1
